# A Case with Mega Cisterna Magna Renal and Ear Anomalies: Is This a New Syndrome?

**DOI:** 10.1155/2013/149656

**Published:** 2013-05-15

**Authors:** Çapan Konca, Bahar Caliskan, Mehmet Ali Tas

**Affiliations:** ^1^Adiyaman Medical Faculty, Pediatrics Department, 02100 Adiyaman, Turkey; ^2^Batman Kozluk State Hospital, Batman, Turkey; ^3^Dicle Medical Faculty, Pediatrics Department, Diyarbakir, Turkey

## Abstract

*Background*. Extrarenal pathologies may be associated with renal position and fusion anomalies. According to the literature, our patient is the first horseshoe kidney case that had mega cisterna magna, arachnodactyly, and mild mental retardation. *Case Report*. A 9-year-old boy admitted because of the myoclonic jerks. He had a dysmorphic face, low-set and cup-shaped ears, arachnodactyly, and mild mental retardation. The patient's laboratory findings were normal except for a mild leucocytosis and hypochromic microcytic anemia. His cerebrospinal fluid was cytologically and biochemically normal. Cranial MRI revealed 1.5 cm diametered mega cisterna magna in the retrocerebellar region. Although there were no significant epileptical discharges in the electroencephalography, there were slow wave discharges arising from the anterior regions of both hemispheres. Because he had stomachache, abdominal ultrasonography was performed, and horseshoe kidney was determined. Abdominal CT did not reveal any abnormalities except the horseshoe kidney. There were not any cardiac pathologies in echocardiography. He had normal 46XY karyotype and there were no repeated chromosomal derangements, but we could not evaluate for molecular and submicroscopic somatic changes. He was treated with valproic acid and myoclonic jerks did not repeat. *Conclusion*. We suggest that the presence of these novel findings may represent a newly recognized, separate syndrome.

## 1. Introduction 

Horseshoe kidney is the most common congenital deformity among renal fusion anomalies, with a male-to-female ratio of 2 : 1. It occurs in 0.25% of the population [1]. Many horseshoe kidneys are asymptomatic and found incidentally. However, they are prone to a variety of complications such as stone disease, ureteropelvic junction (UPJ) obstruction, trauma, infection, and a variety of benign and malignant tumors [2]. Extrarenal pathologies may be associated with horseshoe kidney such as anomalies of the skeletal system, genital anomalies, cardiac anomalies, and digestive disease. Mega cisterna magna (MCM) is described as an enlarged cisterna magna with a normal fourth ventricle and cerebellar hemispheres [3]. The frequency of MCM is 0.4%, and about 62% of the cases have developmental and neurological deficiencies [3]. 

In the literature, there are many reports showing the significant association between renal anomalies and various ear anomalies. Ear and renal anomalies are also components of many multiple congenital anomaly (MCA) syndromes. 

In this report, we describe a boy whose phenotypical features are consistent with MCA syndromes but not specific to any of these syndromes, and we suggest that the presence of these novel findings may represent a newly recognized, separate syndrome. 

## 2. Case Report 

**Figure 1:**
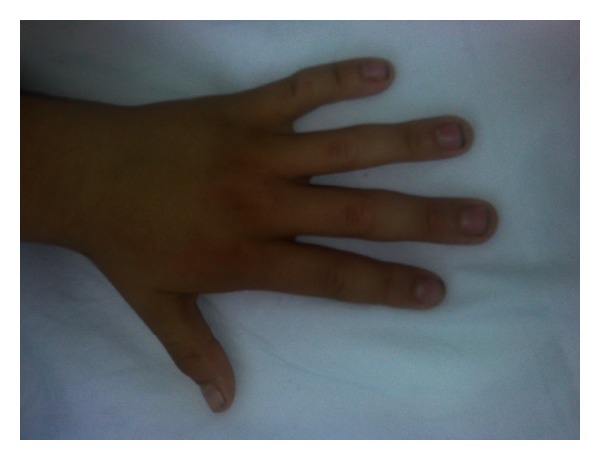
The presence of arachnodactyly.

**Figure 2:**
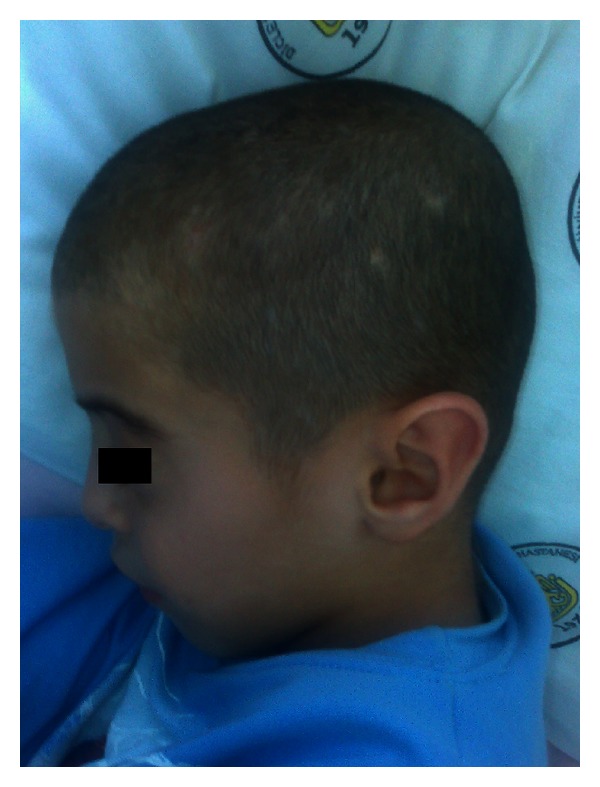
The presence of low-set and cup shaped ears.

**Figure 3:**
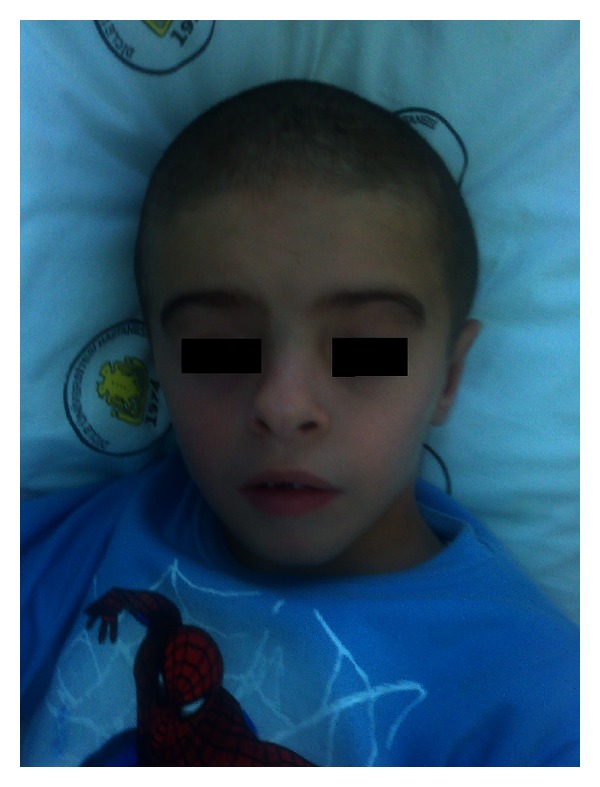
Dysmorphic facial appearance of the patient.

**Figure 4:**
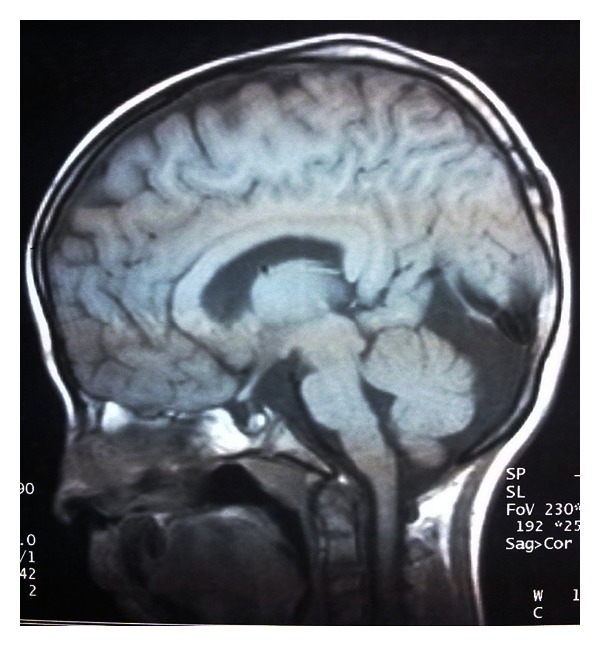
The presence of mega cisterna magna in the retrocerebellar region.

**Figure 5:**
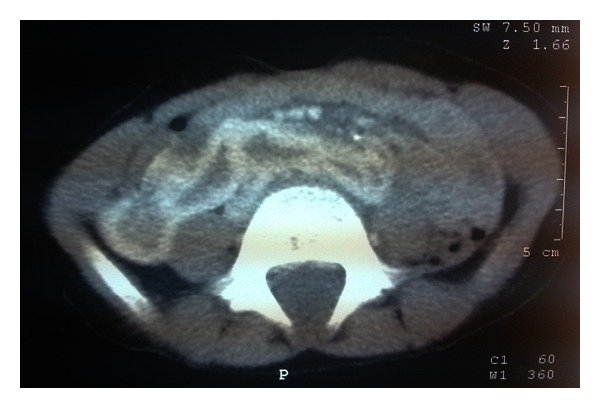
The presence of horseshoe kidney in abdominal ultrasonography.

A 9-year-old boy admitted to the hospital because of the myoclonic jerks of both of his hands and limbs. He had these myoclonic jerks for 2 years, and these movements lasted for 2-3 seconds. The patient was born at term after a normal pregnancy, but because he was born at home his birth weight, birth length and head circumference are unknown. He did not experience any perinatal difficulties. Both parents were healthy, and their examination was normal. There was no history of consanguinity or pregnancy loss. His siblings were healthy with normal psychomotor development, and their examinations were unremarkable. There were no congenital abnormalities in family members. There was no subsequent developmental delay in his motor abilities and cognition. In his physical examination, he had a dysmorphic face (wide forehead, hypertelorism), low-set and cup-shaped ears, arachnodactyly, and mild mental retardation (Figures 1, 2, and 3). Body weight was measured as 26 kg (25–50 percentile) and height as 129 cm (25–50 percentile). The patient's laboratory findings were normal except for a mild leucocytosis and hypochromic microcytic anemia. His cerebrospinal fluid was cytologically and biochemically normal. Cranial MRI revealed 1.5 cm diametered mega cisterna magna in the retrocerebellar region (Figure 4). Although there were no significant epileptical discharges in the electroencephalography, there were slow wave discharges arising from the anterior regions of both hemispheres. Because he had stomachache during his followup, an abdominal ultrasonography was performed, and horseshoe kidney was determined. His abdominal CT did not reveal any abnormalities except the horseshoe kidney (Figure 5). There were not any cardiac pathologies in his echocardiography. He had normal 46XY karyotype, and there were not repeated chromosomal derangements, but we could not evaluate for molecular and submicroscopic somatic changes. He was treated with valproic acid, and myoclonic jerks did not repeat. 

## 3. Discussion 

Horseshoe kidney is the most common congenital deformity among renal fusion anomalies. Extra-renal pathologies may be associated horseshoe kidney such as anomalies of the skeletal system, genital anomalies, cardiac anomalies and digestive disease. The study including 84 patients with kidney position and fusion anomalies have reported that 21 (25.4%) of these patients also had extra-renal pathology such as skeletal, genital and cardiac anomalies, digestive disease, facial malformations and endocrine disorders. As a result, renal-urinary anomalies and structural extra-renal malformations must be evaluated when renal ectopia or horseshoe kidney is diagnosed [4]. 

Congenital asymmetric crying facies (ACF) is considered to be caused by congenital hypoplasia or agenesis of the depressor anguli oris muscle on one side of the mouth [5]. Cardiovascular (ventricular and atrial septal defects, tetralogy of Fallot, and coarctation of the aorta), head and neck (hypertelorism, micrognathia, retrognathia, short frenulum, cleft palate, low-set ears, preauricular tag, mild facial hypoplasia, and hemangioma on the lower lip), musculoskeletal (hypertrophic osteoarthropathy, congenital joint contractures, congenital hip dislocation, and polydactyly), respiratory, gastrointestinal (umbilical and inguinal hernia), central nervous system (cerebral and cerebellar atrophy, mega cisterna magna, mental motor retardation, convulsions, corpus callosum dysgenesis, cranial bone defects, and spina bifida occulta), and genitourinary anomalies (renal hypoplasia and vesicoureteral reflux) are frequently associated with this defect, and all newborns should be carefully examined for this subtle facial sign [5, 6]. Although our patient had musculoskeletal, central nervous system and head anomalies, he did not have hypoplasia of the depressor anguli oris muscle. 

Orocraniodigital syndrome (Juberg-Hayward syndrome) is a rare condition with microcephaly, palatal/lip clefts, and thumb abnormalities [7]. In addition to these abnormalities, low birth weight and short stature are constant findings, features of a widespread skeletal disorders are more variable, and there may be associated renal abnormalities [8, 9]. The severity and extent of congenital anomalies vary among affected individuals. Our patient had horseshoe kidney and arachnodactyly, but as he did not have the cardinal features, we could not diagnose him as orocraniodigital syndrome. 

Multiple congenital anomaly syndromes are a broad spectrum of syndromes involving CHARGE association syndrome, Townes-Brocks syndrome, oculoauriculovertebral spectrum, BOR syndrome, diabetic embryopathy, Treacher Collins syndrome, Nager syndrome, and Miller syndrome [5]. Although our case had most of the specific characteristics of these syndromes, these findings were not enough for diagnosing any of them (Table 1). 

The 22q11.2 deletion syndromes are a group of conditions with a deletion in the long arm of chromosome 22. They share a characteristic spectrum of congenital cardiac defects with wide ranging noncardiac congenital anomalies. They include endocrine abnormalities, immunodeficiency (77%), cardiovascular malformations (75%), craniofacial features, skeletal abnormalities, renal abnormalities (37%), CNS manifestations, and behavior phenotype. Our case had some of these clinical features including low-set ears, horseshoe kidney, mental retardation, and mega cisterna manga. Although our case had arachnodactyly, this feature has not been reported so far in this syndrome.

## 4. Conclusion

In the literature there are many reports showing that renal, head and, central nervous system anomalies can be components of the congenital anomaly syndromes. Since the existing anomalies of our case were not consistent with any of the described syndromes, we suggest that the presence of these anomalies could represent a new and separate syndrome.

## Figures and Tables

**Table 1:** Defined syndromes partially including clinical features of our patient.

Syndromes	Clinical features of our patient						
	Horseshoe kidney	Mega cisterna magna	Ear anomalies	Arachnodactyly	Wide/broad forehead	Hypertelorism	Mental retardation
Del(22q11.2) syndromes	+	+	+	−	−	+	+
Frontonasal dysplasia	−	−	+	−	−	+	+
Dandy-Walker malformation	−	+	−	−	−	+	+
Noonan syndrome	−	−	+	−	+	+	+
Robinow syndrome	−	−	+	−	−	+	−
Wolf-Hirschhorn syndrome	−	−	+	−	+	+	+
Cri-du-chat syndrome	+	−	−	−	−	+	+
Cat-eye syndrome	+	−	+	−	−	+	+
CHARGE association	+	−	+	−	+	−	+
Alagille syndrome	+	−	−	−	+	−	+
Arthrogryposis multiplex congenita	−	−	−	+	−	+	−
VATER (VACTERL) association	+	−	+	−	−	−	−
Fetal alcohol syndrome	+	−	+	−	−	−	+
Fanconi anemia	+	−	+	−	−	+	−
Kabuki syndrome	+	−	−	−	−	−	+
Agnathia	+	−	+	−	−	−	−
Congenital asymmetric crying facies (ACF)	−	+	+	−	−	+	+
LEOPARD syndrome	−	−	+	−	−	+	+

## References

[1] Makita S, Yoshizaki T, Tabuchi N (2009). A case of abdominal aortic aneurysm with horseshoe kidney. *Annals of Thoracic and Cardiovascular Surgery*.

[2] O’Brien J, Buckley O, Doody O, Ward E, Persaud T, Torreggiani W (2008). Imaging of horseshoe kidneys and their complications. *Journal of Medical Imaging and Radiation Oncology*.

[3] Bolduc ME, Limperopoulos C (2009). Neurodevelopmental outcomes in children with cerebellar malformations: a systematic review. *Developmental Medicine and Child Neurology*.

[4] Ubetagoyena Arrieta M, Areses Trapote R, Arruebarrena Lizarraga D (2011). Renal position and fusion anomalies. *Anales de Pediatría*.

[5] Lahat E, Heyman E, Barkay A, Goldberg M (2000). Asymmetric crying facies and associated congenital anomalies: prospective study and review of the literature. *Journal of Child Neurology*.

[6] Caksen H, Odabasi D, Tuncer O (2004). A review of 35 cases of asymmetric crying facies. *Genetic Counseling*.

[7] Hedera P, Innis JW (2003). Juberg-Hayward syndrome: report of a new patient with severe phenotype and novel clinical features. *American Journal of Medical Genetics*.

[8] Bauer SB, Walsh PC, Retik AB, Vaughan ED (2002). Anomalies of the upper urinary tract. *Campbell’s Urology*.

[9] Neven NC, Henry P, Thomas PTS (1981). A case of the orocraniodigital (Juberg-Hayward) syndrome. *Journal of Medical Genetics*.

